# What older adults do with the results of dementia screening programs

**DOI:** 10.1371/journal.pone.0235534

**Published:** 2020-07-01

**Authors:** James E. Galvin, Magdalena I. Tolea, Stephanie Chrisphonte

**Affiliations:** Department of Neurology, Comprehensive Center for Brain Health, University of Miami Miller School of Medicine, Miami, Florida, United States of America; Nathan S Kline Institute, UNITED STATES

## Abstract

**Introduction:**

Alzheimer’s disease and related dementias (ADRD) and mild cognitive impairment (MCI) are often under-recognized in the community. MCI/ADRD screening could offer benefits such as early treatment, research participation, lifestyle modification, and advanced care planning. To date, there are no clear guidelines regarding the benefits vs. harms of dementia screening or whether a dementia screening program could be successful.

**Methods:**

A community-based study was conducted to evaluate an MCI/ADRD screening program and determine what older adults would do with the information. Measures of cognition, physical health, functionality, and mood were collected. Participants met with a health professional, were given screening results with recommendations, and then contacted 60 days later to determine what was done with the results. Logistic regression models were used to build predictive models.

**Results:**

Participants (n = 288) had a mean age of 71.5±8.3y, mean education of 13.3±4.8y, and were 70% female, 67% White, 26% African American, and 48% Hispanic. After 60 days, 75% of participants were re-contacted; 54% shared results with family, 33% shared results with health care providers (HCPs), and 52% initiated behavioral change. Among participants sharing results with HCPs, 51% reported HCPs did not follow-up on the results, and 18% that HCPs did not show any interest in the screening visit or its results. Predictors of sharing results with HCPs were elevated hemoglobin A1C (OR = 1.85;95%CI:1.19–2.88), uncontrolled hypertension (OR = 2.73;95%CI:1.09–6.83), and mobility issues (OR = 2.43;95%CI: 1.93–5.54). Participant behavioral changes included lifestyle modification (58%), social engagement (10%), cognitive stimulation (5%), and advanced care planning (4%). The most significant predictors of sharing with family were better overall mental health (OR = 0.19; 95%CI: 0.06–0.59) and better physical function (OR = 0.38; 95%CI: 0.17–0.81).

**Discussion:**

MCI/ADRD screening was well-received by a diverse community sample. Participants showed interest in sharing the results with their family and HCPs and many attempted behavioral change. While HCPs did not always act on screening results, 25% ordered further testing and evaluation. Efforts need to be directed toward (1) increasing self-efficacy of older adults to discuss screening results with their HCPs, and (2) educating HCPs on the value of early detection of MCI/ADRD. Community dementia screening programs can increase MCI/ADRD detection and improve patient-centered outcomes and medical decision-making.

## Introduction

Alzheimer’s disease and related dementias (ADRD) [1,2] currently affect over 5.7 million Americans and over 35 million people worldwide. The number of ADRD cases is expected to increase as the number of people over age 65 is projected to grow by 62% and the number over age 85 by 84% by the year 2050 [2,3]. Thus, the prevalence, incidence, morbidity, mortality, and social financial burden for ADRD will expand exponentially. Primary care providers are often responsible for the detection, diagnosis, and treatment of ADRD as the number of dementia specialists (neurologists, psychiatrists, and geriatricians) and specialty centers is not sufficient to meet the growing demands [2,3].

Despite these obvious demands for early detection, ADRD and mild cognitive impairment (MCI) [4] are often under-recognized in community practice, with many individuals obtaining a diagnosis in the mild-to-moderate stages of dementia, particularly in older adults from diverse racial, ethnic, geographic, and socioeconomic backgrounds. MCI/ADRD screening would likely increase case identification; however, there are questions as to whether increased screening and case identification have value in the absence of more effective interventions that can improve patient outcomes [5]. Effective public health efforts aimed at secondary prevention (i.e., screening) permit early detection of core elements of disease, hopefully to be coupled with treatment or preventive actions to reduce patient, family, and societal burden of disease [3,6,7].

Screening for MCI and ADRD would likely be most beneficial at the earliest detectable signs of disease, particularly if the detection measures reflect pathology and biomarker changes associated with the earliest stages of ADRD [8,9]. In this way, treatments (both current and future) can be initiated to potentially alter the pathologic cascade [1,4,10] or patients could participate in clinical trials. Patients and families could use this information to be proactive towards brain health and advanced care planning. Furthermore, early dementia recognition could afford clinicians the opportunity to enhance patient adherence by providing information, educational materials, and support to patients and their family caregivers.

However, this premise is not without controversy. To date, there is no definitive recommendation on dementia screening. In 2014, the US Preventative Services Task Force (USPSTF) concluded that the current evidence was insufficient to assess the balance of benefits and harms of screening for cognitive impairment [5]. This document was updated in 2017 to provide a research plan for a systematic review of the evidence. Five Key Questions were developed that need to be addressed before recommendations on cognitive screening could be made: (1) Does screening improve decision-making, patient-, family-, caregiver- or societal outcomes; (2) What is the accuracy of screening instruments; (3) What are the harms of screening; (4) Do interventions improve decision-making and outcomes; and (5) What are the harms of interventions in people with symptomatic disease? [5]

Currently, there are many dementia screening measures available for use, each capturing different aspects of impairment [3]. Some rely on patient performance (e.g., the Montreal Cognitive Assessment or MoCA [11]), while others rely on interviews with collateral sources such as family members who have witnessed change in the patients from their premorbid abilities (i.e., the AD8 [12,13]). More recently, screening measures have been developed that rely on self-report from the patient himself or herself (e.g., the Cognitive Change Index [14]). Some batteries are extensive but time-consuming, making them impractical for use in the context of a busy office setting, while other screening measures are brief, but lack the sensitivity and specificity required to accurately capture those at risk for ADRD [3]. This has led to confusion as to how best to detect MCI and ADRD, what tools to use, and how best to discuss findings with the patient. In a recent report, the Alzheimer Association conducted surveys with 1000 primary health care providers (HCP) and 1954 older adults regarding expectations, benefits, and practices about dementia screening [2]. While 94% of patients saw their HCP in the last year, only 47% discussed memory and only 28% received a memory assessment. This contrasts with 95% of older adults wanting to know about their memory and 51% reporting changes. Although 50% of HCP reported they assess cognition as part of their evaluation, only 40% were familiar with the toolkits available to them. For those that do assess cognition, only 64% informed the patients of the results. This contrasts with more than 90% of HCP reporting there are benefits to dementia screening including advanced care planning and interventions [2]. In prior work, interviewing 1039 older adults in a random digit dialing survey examining intention to screen for dementia, we found that many older adults are not aware their HCP is capable of screening them for cognitive disorders [15]. Although the Medicare Annual Wellness Visit requires some format for cognitive screening, data from our studies and the Alzheimer Association Special Report [2] strongly suggest this is not occurring. Only 20% of Medicare beneficiaries have an Annual Wellness Visit and there are no clear guidelines as to what constitutes a cognitive assessment.

To address questions about whether dementia screening would be acceptable to community dwelling older adults, and what they would do with the results of the screening visit, we completed a community-based dementia screening program on a multicultural sample of 307 older adults.

## Methods

### Participants and study design

Study participants were adults enrolled in cross-sectional clinical research studies between February 2012 and March 2015. A detailed description of these studies was published previously [16,17]. Briefly, community dwelling adults aged ≥55y residing in Manhattan, Queens, and Brooklyn were recruited via collaborations with local community partners, word-of-mouth from prior participants, educational seminars in the community, and from an in-house research registry to enroll in cognitive and functional studies. Screening events were conducted either in the community (libraries, public housing projects, community centers) or at our Research Center. Flyers and announcements were made available to members or residents of the participating community sites. All instruments were translated in Spanish and administered by fluent research staff. Evaluations contained both self-report and performance-based assessments of cognition, physical function, and mood, as well as brief medical screenings for hypertension, diabetes, obesity, frailty, and falls. Written feedback and recommendations were provided to each participant at the end of the visit. Sixty days later, the participants were re-contacted to determine how they used the information provided to them at the screening visit. Exclusion criteria included: age <55y, non-fluency in English or Spanish, and active psychiatric and neurological conditions that could impact physical and/or cognitive performance or could otherwise interfere with participation. The protocol was approved by the NYU School of Medicine Institutional Review Board, Human Research Protection Program and written informed consent was signed by each participant prior to any evaluation.

### Evaluations

#### Demographic information

Demographics, primary language, medical and injury history, medications, alcohol/tobacco/substance use history, co-morbidities, and family history were collected. The Charlson Comorbidity Index [18] was used to measure overall health and medical comorbidities. Socioeconomic status (SES) was calculated using combined education and occupation scores (Hollingshead Four-Factor Index of Social Status [19]) and global questions regarding income.

#### Medical evaluation

A brief physical evaluation was performed during which blood pressure and pulse rate were evaluated and anthropometric measurements by impedance were used to derive body mass index (BMI), basal metabolic rate (BMR) and metabolic age (a comparison of the participant’s BMR against the age-predicted BMR) [20,21]. The Modified Hachinski Scale [22] was used to assess vascular risk. Participants were asked to give global ratings of physical, mental, and emotional health using a 4-point Likert scale (Excellent, Good, Fair, Poor).

#### Physical performance evaluation

The mini-Physical Performance Evaluation (mPPT) [23], Short Physical Performance Test (SPPB) [24], Short Portable Sarcopenia Measure (SPSM) [25], and grip strength by dynamometry were completed to assess physical functioning, mobility, sarcopenia, frailty, and falls risk. As the mini-PPT, SPPB, and SPSM are highly correlated [16,17], only the mini-PPT is reported here since it includes measures of strength, balance, and mobility.

#### Cognitive status

The Montreal Cognitive Assessment [11] was used for a global screen. Additional performance measures included the Mini-Cog [26], and animal naming [27]. The AD8 was used as a self-report measure of cognitive ability [8,12,13]. The Hospital Anxiety and Depression Scale (HADS) [28] was completed to assess mood for orthogonal ratings of depression and anxiety.

### Feedback

At the end of their study visit, all study participants met with a nurse practitioner or licensed social worker to discuss the results of their screening evaluation and receive health recommendations and referrals. Participants were presented with a feedback sheet covering screening results and risk factors for cognitive, physical, and emotional health. Cardiovascular risk indicators included were BMI categorized as underweight, normal weight, and overweight, and blood pressure and heart rate, which were both categorized as low, normal, and high. Metabolic indicators included metabolic age and hemoglobin A1C results. Participants were advised that a metabolic age that exceeds their chronologic age may indicate a need for improving metabolic function by altering dietary patterns and participating in physical activities [29]. Hemoglobin A1C scores were interpreted as indicating either normal metabolic function, being at risk for diabetes (pre-diabetes), or likely having diabetes. Discussed were also results from tests of physical performance including grip strength and mPPT to address physical functionality and falls risk. The inclusion and sharing of the physical exam findings were based on our earlier developmental work that dementia screening would be more socially acceptable if placed in the context of “a healthy body, a healthy mind” and that intention to screen for dementia was predicted by prior preventative health behaviors. [15]

Participants were then informed on their cognitive testing results on the AD8, the MoCA, the Mini-Cog, and animal naming. Participants were presented with the normal range for each test score as well as their score, and informed, when appropriate, that their score fell outside of what is considered normal for individuals of their age. Similarly, participants were informed on their mood ratings from the HADS separately for depression and anxiety; scores were rated as no symptoms, borderline symptoms, or symptoms present.

Given the focus of the feedback on four health domains (general health, cognitive, functional, and mood), a score was created for each domain to indicate presence of impaired function. Impaired cognition was measured as an abnormal score on the AD8 (i.e. ≥2) and/or any of the three performance measures (MoCA: <26; Mini-Cog: <3; Animal Naming: <14). Impaired physical function was defined as poor age- and sex-specific grip strength or mobility impairment (mPPT <12) [21]. The following values were considered as increased risk of general health: BMI ≥25; Systolic Blood Pressure ≥140 mmHg or Diastolic Blood Pressure ≥90 mmHg; Heart Rate ≥100; metabolic age higher than chronologic age; and hemoglobin A1C ≥5.7%. A score of 8+ on the HADS depression or anxiety subscales was considered indicative of impaired mood [28].

After presentation of all the results and a discussion of their interpretation, every participant was encouraged to discuss results with their HCP and family/friends regardless of results. Discussion of positive screening results was encouraged so that appropriate medical action and lifestyle changes could be initiated. Discussion of negative screening results was encouraged so that the participant and their HCP could work to maintain their health and discuss continued health promotion to reduce risk of future disease. If the participant did not have an HCP, the social worker offered to make them an appointment at a local clinic. In addition to discussing the results, participants were given referrals for further cognitive evaluation, mental health, and/or physical therapy services as needed.

Following discussion of the results, the nurse practitioner or social worker offered specific recommendations to the participants on changes in lifestyle practices including daily intake of fruits, vegetables and multivitamins, fish and lean meat consumption, physical activity, cognitive stimulation, social engagement, and future planning tailored to the results of their screening visit. Finally, participants were provided information on local educational and support resources, as well as useful websites (i.e., Alzheimer’s Association, NIH).

### Follow-up interview and outcome measures

Two months after participation in the screening program, participants were contacted by phone and asked to complete a brief, 10-question interview assessing several screening program-related aspects. Several attempts were made to reach participants and when all attempts failed, a note was made in the chart detailing why the follow-up did not take place. The following aspects were assessed during the follow-up, which constitute the outcomes of interest for the current report: (1) Satisfaction with the screening program; and (2) Adherence with screening recommendations. The interview was semi-structured and answers to open ended questions were recorded verbatim and reviewed by the entire study team (neurologist, gerontologist, epidemiologist, nurse practitioner, social worker) and thematic topics (described below) were created. All participant responses were fitted into the thematic topics. Responses were treated with equal weight as recommendations were tailored to the participants’ individual screening results.

*Satisfaction with the information* received during the study was assessed with 5 questions: (1)Where you surprised about the results; (2) How would you rate the information you received; (3) Did you find the information you received useful; (4) How would you rate your overall experience; and (5) Would you recommend participating in research to others? The satisfaction indices were measured on a Likert scale as follows: 1 = excellent; 2 = good; 3 = fair; and 4 = poor. Due to a lack of a normal distribution of responses across these categories, a decision was made to collapse categories as excellent/good and fair/poor. The other two satisfaction indices (i.e. surprised by results and recommend research participation to others) were measured as yes/no variables. Lastly, participants were asked if they had any preferences as to the type of screening methods used (1) interview (i.e., the AD8); (2) performance (i.e., MoCA), or (3) no preference.

*Adherence with recommendations* to share results with their health providers and family members and make changes in lifestyle practices was assessed by asking the following three questions: (1) Did you share the results of your tests with your family; (2) Did you share the results of your test with your physician/health provider; and (3) Did you change any of your habits or activities based on the results of your tests? All three adherence indices were measured as yes/no questions and for the last two, participants were asked to name reasons for not sharing results with their health provider or changing habits. If they shared with health providers or changed habits, participants were additionally asked to identify in an open-ended response what happened. Similarly, if they did not share results with HCP or did not change habits, participants were asked to explain why that happened, in an open-ended response format. Answers were tallied and the following response categories were identified. For the variable measuring the health care provider’s reaction to being informed about the screening results, the following response categories were created: (1) HCP expressed interest but no further action was taken; (2) HCP showed no interest and took no further action; (3) HCP showed interest and ordered further testing; and (4) HCP disagreed with study results. When participants reported not sharing the results with their HCP, several reasons for not sharing results were identified: (1) Participant was not interested in sharing screening results; (2) Participant forgot to mention results to HCP; (3) Participant has not yet had an appointment with their HCP; (4) Participant did not have a HCP; (5) Participant thought HCP would not be interested in hearing the screening results; (6) Participant reported that no feedback information was given to them; and (7) Things got in the way.

Among participants reporting a change in habits/lifestyle based on screening study recommendations the following types of changes were reported: (1) Lifestyle changes (i.e. diet, exercise, stop smoking); (2) Social engagement; (3) Advanced care planning; (4) Cognitive stimulation, and (5) Multiple domain changes (i.e. any combination of the above). When no change in habits was reported, the following reasons were identified: (1) Things got in the way; (2) Participant was not interested in changing; (3) Participant was already doing what was recommended; (4) Participant did not remember that feedback was given; (5) Participant forgot change in habit was recommended; (6) Participant is planning to change habits but has not yet started; and (7) Don’t know.

### Data analysis

To rule out the possibility of underestimation of effects due to a selective drop-out of participants likely to be cognitively impaired and overall unhealthy and therefore unlikely to comply with screening recommendations, we compared those with and without follow-up on socio-demographic, health, cognition, functionality, and mood characteristics with t-tests. The main reasons for not having a follow-up were inability of reaching the subject for follow-up (88.5%) and subject did not remember participating in the screening program (11.5%).

We next assessed the impact of socio-demographics, physical health, cognitive health, and mood on the likelihood of being compliant with study recommendations with multiple logistic regression analysis to allow assessment of independent effects while controlling for the impact of covariates. A forward stepwise method with p = 0.3 for entry and p = 0.35 for removal was used, and the most parsimonious model of factors predicting study outcomes was selected based on adjusted AICs (to account for the small sample size and number of parameters in the model) [30,31]. Step-down Bonferroni correction method was applied to the parsimonious models to account for multiple comparison. Model goodness of fit was assessed with the Hosmer and Lemeshow test and pseudo R^2^ (http://staff.washington.edu/glynn/r2pseudo.pdf) was used as an estimate of magnitude of the overall model effect. In addition, magnitude of effect for each predictor was estimated with OR percent change between individual unadjusted models and the full model.

Finally, the impact of having a newly detected condition or dysfunction on each of the three adherence indicators was assessed with logistic regression. In this set of analyses, participants were categorized into the following groups: controlled disease (self-reported diagnosis and no evidence of the condition), uncontrolled disease (self-reported diagnosis and evidence of condition), and undiagnosed (no self-reported diagnosis and evidence of condition) and compared against those without the condition. For cognitive and mobility dysfunction, likelihood of sharing results and changing habits was compared between the following groups: subjective dysfunction only (self-report but no evidence of dysfunction); objective dysfunction only (no self-report but evidence of dysfunction); subjective and objective dysfunction (both self-report and evidence) and participants with either self-reported or evidence-based dysfunction. Self-reported cognitive dysfunction was based on the AD8, while evidence of cognitive dysfunction was based on impairment in any of the three performance-based cognitive tests used (i.e. MoCA, Mini-Cog, and Animal Naming). Self-reported mobility dysfunction was based on reported mobility and/or movement problems (part of medical history) and evidence-based mobility dysfunction was based on the mPPT using a cutoff of <12. These models were adjusted for significant correlates found in the set of analyses described in the previous paragraph.

## Results

### Sample characteristics

A total of 307 community-dwelling older adults were recruited. Nineteen withdrew from the study after informed consent but before any assessments were administered, leaving a total of 288 participants who contributed data and were therefore included in this study. The participants had a mean age of 71.52±8.3y (range: 55–100) and a mean education of 13.3±4.8y (range: 0–20). The sample was 70.5% female; 66.9% White and 25.9% African American, with 47.7% of the sample reporting Hispanic ethnicity (comprised of 36.1% South American, 33.1% Puerto Rican, 11.3% Dominican, 10.6% Mexican and Central American; 3.8% Cuban, and 5.3% Other/Not Specified). English was the primary language in 58.2% of the sample. The participants were largely independent (89.6%), living alone (49.7%) in a single-family residence or apartment (96.8%). The mean Hollingshead Index of Social Status was 40.8±19.1 (range 11–77) supporting a wide range of SES.

The mean AD8 score was 1.9±1.9 (range: 0–8), mean MoCA score was 22.3±5.3 (range: 1–30) and mean Mini-Cog score was 2.6±1.3 (range: 0–4) supporting a wide range of cognitive performance. The mean mPPT score was 11.7±2.9 (range: 0–16) and the mean Charlson Comorbidity Index was 5.9±2.1 (range: 2–13) supporting a wide range of physical functionality and comorbidities. The mean HADS-anxiety score was 5.6±3.7 (range: 0–19) and mean HADS-depression score was 5.6±3.9 (range: 0–21) supporting a wide range of mood states. The participants provided self-ratings of good to excellent physical health (67.4%), mental health (76.2%), and emotional health (68.2%) at the time of their screening visit.

After 60 days, we were able to contact 74.7% of participants for their follow-up interview, however 3 of these did not remember having participated in the screening program and therefore had no valid data leaving a total of 212 (73.6%) participants with a valid follow-up. Participants without a follow-up (N = 76) were more likely to be White (p = 0.04) and Hispanic (p = .002) and were less likely to have baseline anxiety (p = 0.04) but did not differ from participants with follow-up in terms of age, sex, SES, health measures, functionality, or cognition (Table 1).

**Table 1:** Participant characteristics by follow-up status.

Variable (Range of scores)	Had follow-up (N = 212)	Did not have follow-up (N = 76)	P value
**Age** (range: 55-100yrs.)	71.7±8.3	70.9±8.1	0.45
**Sex, Female** (%)	70.3	71.1	0.90
**Non-White Race,** (%)	37.7	19.4	0.04
**Hispanic,** (%)	42.2	63.2	0.002
**Social status** (range: 11–77)	40.8±18.8	40.7±20.3	0.96
**Morbidity**^α^ (range:2–13)	6.0±2.2	5.7±1.9	0.27
**Mobility**^β^ (range: 0–16)	11.6±3.0	12.0±2.8	0.38
**AD8** (range: 0–8)	1.8±1.9	2.1±2.1	0.33
**Animal Naming** (range: 1–36)	16.3±6.1	16.3±5.3	0.96
**MoCA** (range: 1–30)	22.5±5.1	21.7±6.1	0.29
**Mini-Cog** (range: 0–4)	2.6±1.3	2.6±1.3	0.91
**Depression** (range: 0–21)	5.5±4.0	5.8±3.6	0.69
**Anxiety** (range: 0–19)	5.9±3.7	4.8±3.6	0.04

Abbreviations: MoCA = Montreal Cognitive Assessment

^α^Morbidity was measured with the Charlson Morbidity Index;

^β^Mobility was measured with Mini PPT; Differences between the two groups were tested with chi square for categorical variables and t test for interval variables.

### Experience with screening visit

Most participants had a positive experience with the screening program (92.7%), finding the information they received useful (92.2%) and of good/excellent quality (91.8%), and would recommend participation in similar research studies to others (95.0%). Many participants (60.1%) were not surprised by the results of the screening visit. There was no preference between interview and pencil-and-paper screening tests by sex, race, ethnicity, or SES (data not shown).

### Impact of socio-demographics, physical health, cognitive health, and mood on compliance with screening recommendations (Table 2)

**Table 2:** Correlates of sharing screening results with family and HCP and changing habits.

		N(Mean±SD)/N (%)	Sharing with family	Sharing with HCP	Changing habits
			OR (95%CI) % change in OR/adjusted p value ^γ^	OR (95%CI) % change in OR /adjusted p value ^γ^	OR (95%CI) % change in OR /adjusted p value ^γ^
	N (full/parsimonious model)		79	76	55
Physical health	Mini PPT	206 (11.6±3.0)	-	0.803 (0.644–1.001) -0.14/0.333	-
	Charlson Index	177 (6.1±2.2)	-	-	0.618 (0.416–0.919) -0.25/0.116
	Hemoglobin A1C	80 (6.3±2.0)	1.435 (0.958–2.147) 0.09/0.239	1.853 (1.193–2.879) 0.36/0.048	2.192 (1.154–4.164) 0.88/0.116
	Grip strength	207 (51.0±21.3)	-	-	-
	Mean arterial pressure	208 (95.5±12.0)	0.939 (0.897–0.990) -0.06/0.093	1.000 (0.947–1.056) -0.003/1.000	-
	Fair/poor physical health ^ʢ^	66 (31.5%)	-	-	-
Mental health	MoCA	208 (22.5±5.1)	1.092 (0.980–1.216) 0.01/0.239	1.078 (0.941–1.235) 0.06/0.845	0.826 (0.679–1.005) -0.17/0.169
	Animal naming	184 (16.3±6.1)	-	-	1.113 (0.953–1.300) 0.06/0.177
	Mini Cog	192 (2.0±1.0)	-	2.122 (0.943–4.777) 0.50/0.345	-
	Fair/poor mental health ^ʢ^	47 (22.2%)	0.194 (0.064–0.590) -0.71/0.023	-	-
Emotional health	Depression	203 (5.5±4.0)	-	-	-
	Anxiety	203 (5.9±3.7)	-	-	-
	Fair/poor emotional health ^ʢ^	70 (33.2%)	-	1.805 (0.834–3.908)0.46/0.537	2.144 (0.916–5.017) 1.12/.0169
Socio-demographics	Age	212 (71.7±8.3)	-	0.920 (0.847–0.999) -0.06/0.333	1.142 (1.023–1.275) 0.20/0.116
	Female	149 (70.3%)	-	-	-
	Non-Hispanic White	75 (35.6%)	1.899 (1.010–3.570) 0.57/0.186	-	-
Goodness of fit^£^	λ^2^ ^¥^	-	4.298	14.574	5.606
	P value	-	0.829	0.068	0.587
Model overfit testing	AIC		60.37	50.39	70.85
	Adjusted AIC ^Ɛ^		61.57	52.89	72.67
	Pseudo R^2^		0.209	0.209	0.032

^γ^ Parsimonious models obtained with forward stepwise logistic regression with the following parameters: p = 0.3 for entry and p = 0.35 for removal. To account for multi comparison, p values (in the presented parsimonious models) were adjusted with the step-down Bonferroni method. Unadjusted and adjusted p values are presented.

^£^ From Hosmer and Lemeshow Goodness of Fit Test—p values >0.05 indicate good fit.

^¥^ Chi square statistic.

^λ^ Adjusted for sample size and number of variables in the model.

^ʢ^ Self-reported health measure. Pseudo R^2^ estimated with an online calculator available from http://staff.washington.edu/glynn/r2pseudo.pdf.

#### Sharing results with family

A total of 115 representing 56% of participants reported sharing results with their family. Individuals with objective cognitive deficits on MoCA (48.9% vs. 68.6%, p = .007) or Animal naming (40.9% vs. 62.5%, p = .005) but not on AD8 (54.5% vs. 57.1%, p = 0.698) were less likely to share results with their family than those without deficits. Participants were not more likely to share results of medical, mood, or functional screening with their family, whether or not they had cognitive deficits detected (data not shown). The most parsimonious model predicting sharing with family included a trend for lower mean arterial pressure (OR = 0.94, 95%CI: 0.89–0.99, p = 0.093) and higher self-perceived mental health (OR = 0.19, 95%CI: 0.06–0.59, p = 0.023). An increase of 71% in the effect of self-perceived mental health (OR) was found between the unadjusted and full models. Minority status lost its significance (OR = 1.90, 95%CI: 1.01–3.57, p = 0.046) when Bonferroni correction was applied to the parsimonious model. Model fit statistics demonstrated goodness of fit (χ^2^ = 4.30, p = 0.83; pseudo R^2^ = 0.209).

#### Sharing results with HCP

**Fig 1:**
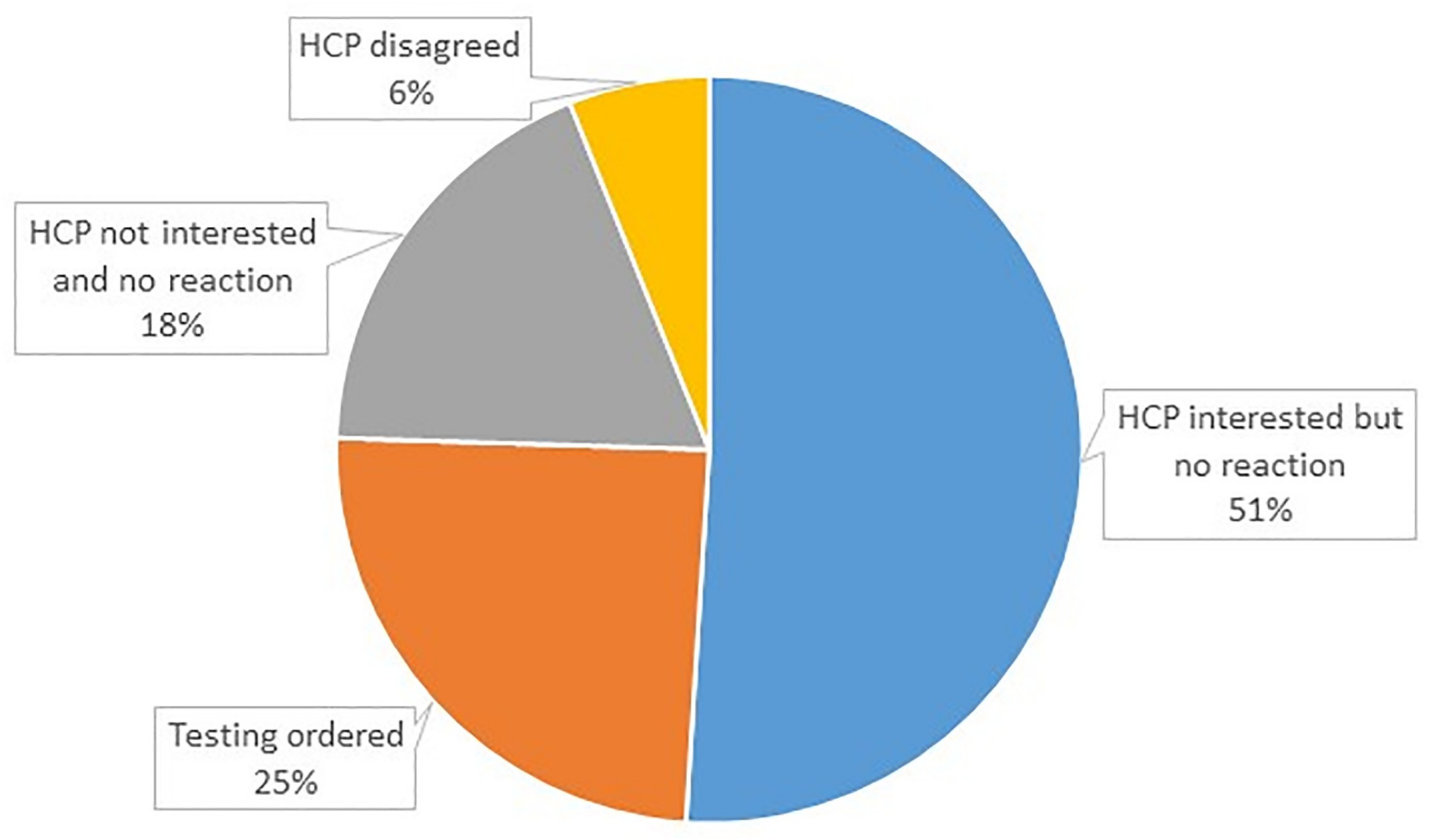
Health care provider response to sharing screening results. Thirty-three percent of participants shared the results of their screening with their health care providers. When asked to describe what the providers response was, 25% reported that their providers ordered additional tests and initiated a work-up to establish a diagnosis. Participants reported that 6% of providers disagreed with the results of the screening visit and took no further action, while 18% of providers were not interested in reviewing the results of the screening visit. Participants reported that 51% of providers discussed the results of the screening visit with the participant but took no further action with the proportion tending to be higher in those who were found to be cognitive impaired based on both objective and subjective tests compared to those who were not impaired (60.5% vs. 18.2%, p = 0.050).

A total of 64 (9 were excluded because they did not have an HCP) representing 32% of participants reported sharing results with their HCP. Of these, 15 did not provide a response resulting in 49 participants with valid responses, of whom, 51% reported their HCP seemed interested but did not follow-up on the results (did not significantly differ by cognitive impairment status: 60.5% (impaired) vs. 18.2% (not impaired), p = 0.050), 25% ordered further tests, and 18% did not show any interest in the screening results (Fig 1). HCP response did not vary by age, sex, race, or ethnicity of participant. Of those not sharing results with their HCP, 43% reported they had not yet made an appointment, 19% stated a lack of interest in sharing the results, and 17% forgot to mention the results. Sharing with HCP was best predicted by a combination of high hemoglobin A1C score (OR = 1.85; 95%CI: 1.19–2.88) and younger age (OR = 0.92, 95%CI: 0.85–0.99). Model fit statistics suggest good fit (χ^2^ = 14.57, p = 0.07, pseudo R^2^ = .0.209). After adjustment for multiple comparison was applied, significance was retained for hemoglobin A1C (p = 0.048) only, whose adjusted effect increased by 36% compared to the unadjusted model.

#### Change in behavior

**Fig 2:**
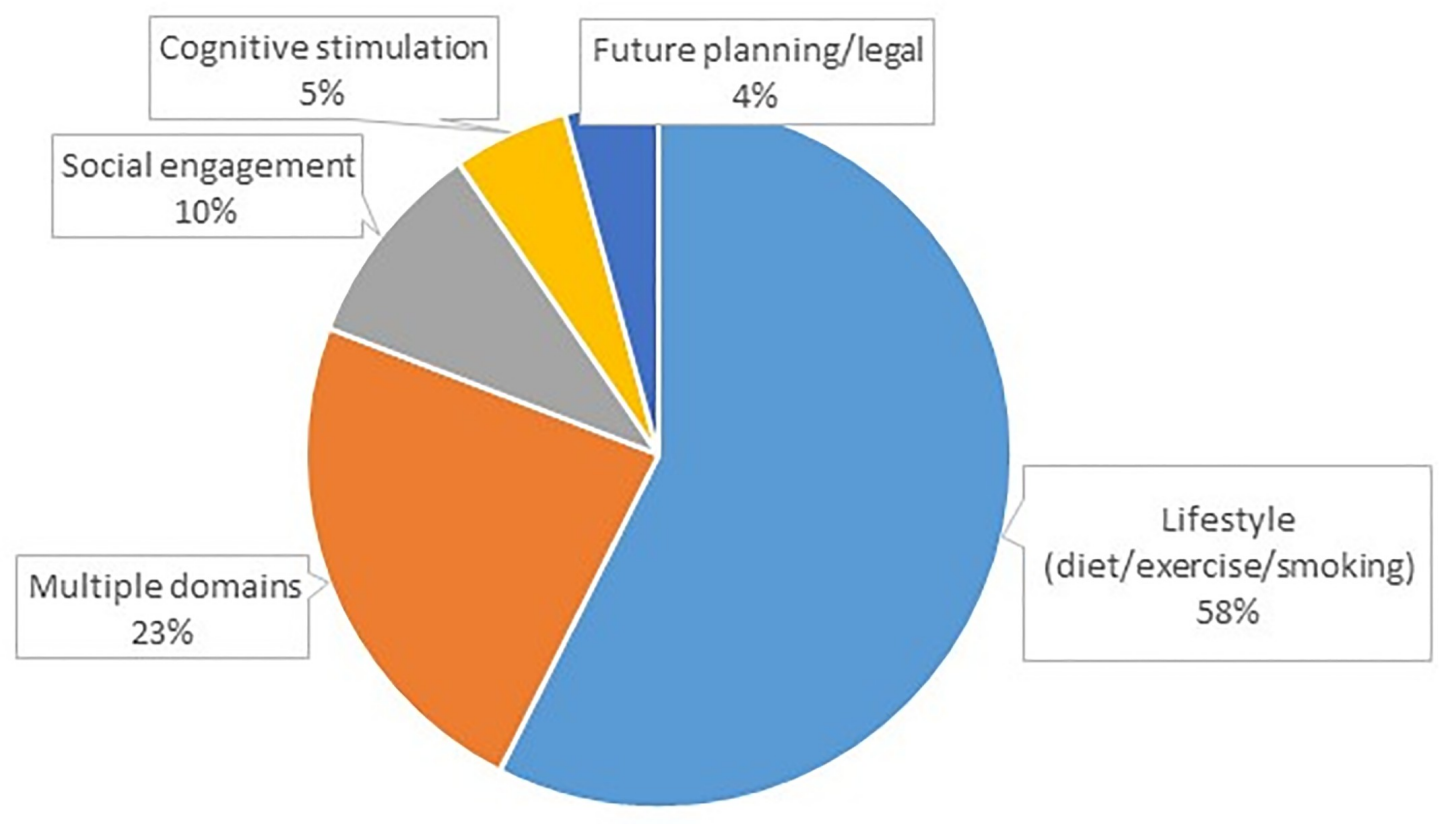
Behavioral changes reported by participants following dementia screening. About half of participants (49%) initiated some healthful behavioral change after receiving the results of the screening visit. The majority of participants (58%) made lifestyle changes including changing their diets, increasing exercise and smoking cessation. Other activities included increasing social engagement (10%), increasing cognitive stimulation (5%) and initiating advanced care planning (4%). Nearly a quarter of participants initiated behavioral changes in multiple domains.

A total of 100 (49%) participants reported a change in behavior following the screening visit. Lifestyle changes (e.g., diet, exercise) were the single most common type of behavioral change reported (58.0%) followed by increasing social engagement (9.6%), cognitive stimulation (5.3%), and advanced care planning (4.3%); 23.4% of participants reported making changes in two or more domains (Fig 2). Reasons reported for not changing habits included no need for change (“Already doing what was recommended”; 37.4%), things got in the way (26.4%), planning to change habits in the future (13.2%), and not interested (12.1%). The likelihood of changing habits was best predicted by lower comorbidities (OR = 0.62, 95%CI: 0.42–0.92), higher A1C (OR = 2.19, 95%CI: 1.15–4.16), and increased age (OR = 1.14, 95%CI: 1.02–1.28). Model fit statistics were split with χ^2^ = 5.61, p = 0.29 but low pseudo R^2^. Bonferroni correction reduced the impact of these factors (comorbidities from p = 0.017 to p = 0.116; hemoglobin A1C from p = 0.016 to p = 0.116; age from p = 0.018 to p = 0.116).

### Impact of newly detected conditions/dysfunction on compliance with screening recommendations (Table 3)

**Table 3:** Disease status and subjective versus objective cognitive and mobility function as correlates of adherence with screening recommendations.

		N (%)	Sharing with family (N = 115)	Sharing with HCP (N = 64)	Changing habits (N = 100)
			OR (95%CI) ^γ^	OR (95%CI) ^€^	OR (95%CI) ^Ɛ^
Hypertension	No (0)	61 (29.8%)	ref	Ref	ref
	Controlled (1)	50 (24.4%)	1.387 (0.618–3.113)	2.128 (0.864–5.237)	1.497 (0.373–6.017)
	Undiagnosed (2)	34 (16.6%)	1.256 (0.521–3.029)	2.186 (0.838–5.705)	0.415 (0.082–2.089)
	Uncontrolled (3)	60 (29.3%)	1.340 (0.593–3.029)	**2.730 (1.091–6.829)**	1.164 (0.357–4.829)
	Goodness of fit	λ^2^ ^¥^ (p value)	13.629 (0.092)	8.133 (0.421)	4.886 (0.844)
Diabetes	None (0)	23 (28.89%)	ref	Ref	ref
	Controlled (1)	3 (3.8%)	- ^£^	- ^£^	- ^£^
	Undiagnosed (2)	44 (55.0%)	1.728 (0.606–4.926)	0.880 (0.258–3.000)	0.526 (0.155–1.785)
	Uncontrolled (3)	10 (12.5%)	- ^£^	- ^£^	- ^£^
	Goodness of fit	λ^2^ ^¥^ (p value)	8.493 (0.291)	8.493 (0.291)	3.806 (0.924)
Cognitive dysfunction	None (0)	35 (16.5%)	ref	Ref	ref
	Subjective (1)	26 (12.3%)	0.750 (0.248–2.264)	0.938 (0.304–2.890)	3.229 (0.471–22.127)
	Objective (2)	73 (34.4%)	0.432 (0.180–1.040)	0.629 (0.253–1.568)	3.175 (0.550–18.314)
	Subjective and objective (3)	78 (36.8%)	0.502 (0.206–1.223)	0.982 (0.792–2.443)	1.903 (0.324–11.196)
	Goodness of fit	λ^2^ ^¥^ (p value)	8.741 (0.365)	7.039 (0.533)	5.586 (0.694)
Physical dysfunction	None (0)	71 (34.0%)	ref	Ref	ref
	Subjective (1)	18 (8.6%)	0.456 (0.152–1.370)	**3.160 (1.030–9.695)**	0.270 (0.026–2.823)
	Objective (2)	61 (29.2%)	**0.375 (0.174–0.808)**	1.539 (0.672–3.525)	0.820 (0.261–2.572)
	Subjective and objective (3)	59 (28.2%)	0.706 (0.320–1.555)	**2.430 (1.929–5.542)**	0.403 (0.114–1.423)
	Goodness of fit	λ^2^ ^¥^ (p value)	5.309 (0.724)	13.613 (0.092)	4.508 (0.809)

^£^Not included due to small numbers.

^γ^ Models for hypertension, diabetes, and mobility dysfunction were adjusted for age and MoCA while model for cognitive dysfunction was adjusted for age.

^€^ Models were adjusted for mini PPT and age, while model for physical dysfunction was adjusted for age.

^Ɛ^ Models adjusted for hemoglobin A1C and age.

^¥^ Chi square statistic.

We assessed the impact of undiagnosed/uncontrolled health conditions (i.e. hypertension and diabetes) or cognitive and mobility dysfunction on adherence with screening recommendations. As these factors were found to be moderately-to-highly correlated to variables assessed in Table 2 (r = -0.58, p<0.001 for cognitive dysfunction-MoCA; r = -0.59, p<0.001 for physical dysfunction-mini PPT; r = 0.42, p<0.001 for diabetes-hemoglobin A1C; r = 0.60, p<0.001 for HTN-mean arterial pressure), a decision was made to analyze them separately. We found objective mobility dysfunction (OR = 0.38, 95%CI: 0.17–0.81) to be associated with decreased likelihood of sharing screening results with family. Participants with a prior diagnosis of hypertension were more likely to share the results of their cognitive screening with their HCP, particularly when their disease was uncontrolled (OR = 2.73, 95%CI: 1.09–6.83). Self-reported mobility dysfunction increased the chances of sharing with HCP, whether or not objective evidence was found (OR = 2.43, 95%CI: 1.93–5.54 and OR = 3.16, 95%CI: 1.03–9.70, respectively). In addition, none of conditions assessed was found to further predict likelihood of changing habits.

## Discussion

Our main objective in this study was to assess a community-based MCI and ADRD screening program and participant adherence with recommendations for clinical follow-up, sharing results with family and HCP, and initiating lifestyle changes. We found that screening for MCI and ADRD was feasible in a diverse community sample and participants were satisfied with the program and discussion of their results. The participants showed some interest in sharing the results with their family and HCP and about half attempted some form of behavioral change based on their results.

We found that participants reported high levels of satisfaction with the information received during the screening program, with their overall experience, and expressed an interest in participating in further research, however adherence with the provided recommendations was lower than expected. A lack of perceived urgency to, interest in, or need to follow recommendations were the top reasons for the low rates of adherence in our study. Characteristics likely to promote adherence were also identified including better metabolic function, better perceived mental health, and better control of hypertension. Those reporting a change in habits following screening feedback were more likely to make lifestyle changes (i.e. diet, exercise, smoking) (58%) or changes in multiple domains combining lifestyle changes with social engagement, legal/planning, and/or cognitive stimulation (23%). Our findings could be interpreted to suggest that older adults attending community-based dementia screening programs are more likely to be adherent to screening recommendations to share with their HCP when results indicate poor physical-related outcomes including poor glycemic control and physical dysfunction. Those cognitively well are more likely to remember to share and inform their family about their participation in the screening program. Alternatively, participants may have selectively decided to share results when physical rather than cognitive problems were found due to stigma related to a cognitive impairment/dementia diagnosis [32].

Family is a major source of emotional and financial support to many older adults and particularly to minorities. In the Latino culture, for example, medical issues are discussed and decisions are made with input from the larger family [33]. We found that, in unadjusted analyses racial and ethnic minorities were more likely to share screening results compared to Whites in our study, however this relationship was no longer significant after Bonferroni correction.

### The potential value of dementia screening

There is great debate regarding the value of dementia screening. The USPSTF recently published an updated statement reaffirming a prior conclusion that there is insufficient evidence to support screening for MCI and ADRD [5]. Other investigators have taken similar stances [34–36] arguing that there is a low prevalence of dementia in the general populations, the clinical course of MCI and ADRD is not altered with currently available interventions, and voicing concerns about possible stigma (employment, insurance), psychological reactions by patients (depression, anxiety), and questions as to whether health systems are sufficiently prepared to screen millions of people [34–38]. Questions have also been raised as to whether medical decision making is altered by early detection for patients, caregivers, or health professionals [5]. There may be important differences regarding dementia screening in other countries that are dependent on cultural norms, resources, and availability of clinical services [39,40].

The alternative view of this argument is that patients have the right to know if they have a medical condition so that they can take whatever actions are currently available, be proactive in lifestyle modifications and advanced care planning, and possibly participate in clinical trials to test new therapies. Dementia screening has been found to be generally acceptable [41–43] and feasible in primary care settings [44,45]. There is evidence that proactive screening in settings such as the hospital can reduce length of stay, reduce delirium, and decrease 30-day readmission rates [46–48]. Screening for dementia in the Emergency Department setting may reduce diagnostic uncertainty and help with medical decision-making [49].

In a Special Report published by the Alzheimer’s Association [2], nearly all HCP and 80% of older adults thought that cognitive assessments are beneficial yet less than half of older adults are ever evaluated. This suggests an important disconnect regarding expectations of MCI and ADRD screening between providers and patients. Patients appear to expect their providers to recommend and perform screening, while providers appear to be waiting for patients to complain about the memory and ask to be tested [2]. ADRD screening is ideally suited for the primary care setting as the providers typically have long standing relationships with patients, have more readily available appointment slots, are already following other chronic conditions, and can provide continuity of care. In particular, the Medicare Annual Wellness visit was designed to take advantage of this setting and has assessment of cognitive function as a requirement. However only 20% of Medicare beneficiaries have an Annual Wellness Visit, and there are no clear guidelines as to what constitutes a cognitive assessment. In the Alzheimer Association report, PCP reported that 50% assess cognition as part of their evaluation but only 40% are familiar with the toolkits available to them [2]. For those that do assess cognition, only 64% informed the patients of the results [2]. In the current study, we found that even when presented with the results of screening, 75% of HCP did not act on the information, this was particularly true for individuals who screened negative for dementia but were given information on preventive lifestyle changes. These results mirror other reports of low rates of new physician action on screening results for 748 patients attending primary care clinics [50], although in this study physician action was limited to the severe cognitive impairment cases (Mini-Cog score 0/5). It is however encouraging that in 25% of cases, HCP did follow-up on the screening results, ordered further testing, and therefore initiated the process of obtaining a diagnosis. Further studies should be designed to determine clinician-related factors that promote dementia diagnosis and treatment once impairment is detected by screening programs. There is clearly a need for more research to develop usable guidelines and recommendations not only regarding to when to screen, but how to do it, what tools to use, which patients to assess, how to discuss the results of screening, and what to do with a positive screen. There are currently toolkits available from the Alzheimer’s Association, National Institutes of Health, Gerontological Society of America, American Academy of Family Physicians, and other professional associations. The Alzheimer’s Association Special Report suggests that only 40% of HCP are familiar with these toolkits [2].

### Does screening lead to behavioral change?

Although a small sample size minimized our ability to clearly identify factors that explain habit changes in our sample, higher A1C, older age, and lower burden of disease may be considered as potential players in whether individuals take action to change habits. Although it has been proposed that initiation of behavioral change in older adults may be more difficult [51] due to lack of motivation [52,53] and a tendency to be less open to new experiences and to avoid novelties [54], we found some evidence that behavioral modification can be achieved in older adults especially when faced with the prospect of being at increased risk of diabetes. If replicated in larger studies, this would support the notion that lifestyle modification interventions may be successful in helping older adults move further in the behavioral change process by advancing from pre-action to action stages [55]. In addition, further research is needed to determine whether healthy behavioral change initiated in response to feedback provided as part of a screening program can be maintained long-term.

In studies of intention to have dementia screening, several constructs were found to be predictive [15,56] including increased knowledge of disease and consequences, perceived susceptibility, self-efficacy, and participating in other preventative health behaviors (e.g., eye exams, cancer screening). The results of the present study suggest that following actual screening, adherence with recommendations will require directed educational programming for both the general population on how to discuss findings with families and HCP, and for health professionals on what to do with the results given to them.

### Does the presence of co-morbid condition impact adherence?

Our expectation was that sharing with family/HCP and changing habits may be enhanced by a newly ‘found’ condition (reported in the feedback were blood pressure, hemoglobin A1C, muscle strength, mobility, mood, and cognition). However, we found that adherence was higher in those who were aware of their conditions, particularly if they were not controlled. For example, the odds of sharing with HCPs were 3-fold in those with uncontrolled hypertension. Similarly, while we expected that adherence would be higher in those not aware of their cognitive or mobility dysfunction, we found that those who perceived their mobility as being impaired were more likely to share screening results with their HCP than those physically normal whether or not objective evidence was also found. In contrast, those who did not think their physical functionality is impaired but performance-based testing confirmed impairment were less likely to share with family. These findings suggest that adherence was higher in those for whom the conditions investigated in the screening and reported in the feedback were known or suspected. Finding strategies to improve adherence especially among those with newly detected conditions is important as they are the ones to benefit most from screening programs.

### Limitations

Our findings should be interpreted in light of study limitations. First, while our study population was racially and ethnically diverse, some racial groups such as Asian Americans and American Indians were underrepresented, precluding the generalizability of our results to these populations. Second, older adults attending community dementia screening events may be more motivated than others to seek early cognitive evaluation. The number of people that participated (n = 307) are the number of people that approached us to participate. Other than age, there were few exclusion criteria. There is no way to know the number of people who saw the flyers and chose not to participate. This may lead to a selection bias related to individuals who did not want to participate. There is also no way to know who did not see the flyers and therefore did not participate because they were unaware of the event. Third, our short follow-up timeframe (i.e. 60 days following screening) may not have allowed enough time for participants to see their HCP to discuss the screening results, which may have led to underestimation of this index of adherence. However, given the consistent trend for low adherence across all three indices, the impact of the short follow-up is likely minimal. On the positive side, this was a large community-based dementia screening program that incorporated multiple domains and participants received feedback on each domain and referral to health services available in their communities. The tremendous diversity of the sample reflects the population of New York City and increases the generalizability of the findings.

### Future directions

One of the key questions raised by the USPSTF was whether dementia screening could alter medical decision making for patients and health professional. Prior studies suggest that these programs may offer the advantages of greater levels of standardization, comprehensiveness, and efficiency in testing as well as provision of educational resources to patients/family [57] usually unavailable in the primary care office. These advantages can translate into referrals for formal diagnosis at earlier disease phases compared to other methods including physician referral. In a retrospective study of patients with AD attending an outpatient memory clinic, those referred by a community memory screening program had a shorter duration of disease, lower frequency of psychosis, and higher cognitive scores compared to patients referred through other venues including their physician, even after an 18-month lag between the screening and diagnostic visits [57]. In addition, community dementia screening may reduce dementia care costs in the long run, especially if formal diagnostic and therapeutic interventions are initiated within a year from screening [44]. Other studies support that screening and case ascertainment may have benefits for hospital systems [46–49].

One of our main objectives was to evaluate if a community-based dementia screening program could work. This is similar in thinking to other community-based screening programs (e.g., hypertension, breast cancer)–go out into the community, identify cases, and then refer for medical care and/or services. This would not necessarily replace the role of the HCP but instead complement it, particularly in areas where access to primary care is limited (e.g., minorities, under- or uninsured, rural areas). We proposed one form of screening—combining basic medical screening with dementia screening to remove stigma and increase willingness to participate. We also largely did this in the community, putting all our equipment in Pullman luggage and taking cabs, subways, and buses to community centers, libraries, churches, and public housing. Other investigators have proposed other screening programs through primary care practices or reviews of electronic medical records. In reality, it is probably a combination of all. Further research will be needed to determine this.

### Concluding remarks

While this study cannot answer all the questions raised by the USPSTF regarding potential harms and benefits, it does provide supporting evidence that dementia screening is well accepted by diverse communities, does not appear to cause any undue psychological harm, and may improve chances for further evaluation as at least 50% of those screened to share results with their family and make some lifestyle changes, and at least a third of participants shared the results with their HCP. Educational programs to increase awareness of the important role screening plays in dementia care may be more effective if caregivers/family are available to help move the affected person along the dementia care process. However, this can be achieved only through patient-clinician collaboration, which we found to be lacking and was reflected in the Alzheimer’s Association Special Report [2]. Efforts need to be directed toward (1) increasing self-efficacy of older adults to discuss screening results with their HCPs, and (2) educating HCPs on the value of early detection of MCI/ADRD. The likelihood of improved adherence and follow-up with screening results should be investigated in the context of community dementia/health screening programs that incorporate additional elements supporting participants in their quest for early diagnosis. Community dementia screening programs can increase MCI/ADRD detection and improve patient-centered outcomes and medical decision-making.
